# Social influence on selection behaviour: Distinguishing local- and global-driven preferential attachment

**DOI:** 10.1371/journal.pone.0175761

**Published:** 2017-04-13

**Authors:** Xue Pan, Lei Hou, Kecheng Liu

**Affiliations:** 1 Informatics Research Centre, Henley Business School, University of Reading, Reading RG6 6UD, United Kingdom; 2 Data Science and Cloud Service Research Centre, Shanghai University of Finance and Economics, Shanghai 200433, China; East China University of Science and Technology, CHINA

## Abstract

Social influence drives human selection behaviours when numerous objects competing for limited attentions, which leads to the ‘rich get richer’ dynamics where popular objects tend to get more attentions. However, evidences have been found that, both the global information of the whole system and the local information among one’s friends have significant influence over the one’s selection. Consequently, a key question raises that, it is the local information or the global information more determinative for one’s selection? Here we compare the local-based influence and global-based influence. We show that, the selection behaviour is mainly driven by the local popularity of the objects while the global popularity plays a supplementary role driving the behaviour only when there is little local information for the user to refer to. Thereby, we propose a network model to describe the mechanism of user-object interaction evolution with social influence, where the users perform either local-driven or global-driven preferential attachments to the objects, i.e., the probability of an objects to be selected by a target user is proportional to either its local popularity or global popularity. The simulation suggests that, about 75% of the attachments should be driven by the local popularity to reproduce the empirical observations. It means that, at least in the studied context where users chose businesses on Yelp, there is a probability of 75% for a user to make a selection according to the local popularity. The proposed model and the numerical findings may shed some light on the study of social influence and evolving social systems.

## Introduction

Social influence plays a notable role in various domains of human behaviour, such as the interpersonal health [1, 2], political attitude [3, 4] and cultural product consumption [5, 6]. Especially in the common context when selecting from countless objects, for example books, movies, restaurants etc., people frequently look at others’ decisions [7–9].

It is very easy nowadays for people to access the information of objects such as qualities, ratings, popularities or even previous consumers’ feedbacks from the mass media. Particularly, in many online systems, bestseller lists or highest-rated object lists are generally available for users to refer to. Those information aggregating the choices and opinions of the whole population of the system, can be recognised as the **global information** which has long been argued to be the key reference for human selection behaviour [5, 8, 10, 11] leading to the ‘rich get richer’ phenomenon. A good example is the event that, two scholars secretly purchased 50,000 copies of their newly published book which consequently made the bestseller list. Then the book sold very good despite mediocre reviews, and was remained as a bestseller [10]. Inspired by the preferential attachment mechanism in many social and technical systems [12, 13], various models [14–16] have been developed to describe the global-based influence, where the popularity is the key indicator of an object’s attractiveness for future selections.

Another mainstream of the social influence research believes that people in the same social group act similarly to each other [17, 18] since individuals are always engaged in group activities. Such source of the influence which can be regarded as the **local information**, also drives the human selection behaviour, i.e. people tend to select what their friends selected [19, 20]. It has long been argued that, objects are similar to viruses and ideas that could spread in the social network from an individual to his/her friends through the frequent interactions [21, 22]. Accordingly, the local-based social influence is also recognised as the social contagion phenomenon [23, 24].

Despite numerous evidences have been found that, both the global-based influence and the local-based influence can drive human behaviours, few of the previous investigations distinguish and compare these two sources of social influence. Onnela and Reed-Tsochas argued that it is important to distinguish the local and global sources of social influence [25]. However, the method they applied in their study, namely the fluctuation scaling, though successfully revealed the emergence of the social influence in terms of popularity, could not efficiently distinguish the local- and global-based influence. Lee *et al*. recently discussed the crowd’s (global-based) and friends’ (local-based) influence over users’ behaviours of rating movies [26]. For a specific movie that has already been selected by a user, his/her rating on it is very likely to be influenced by the previous one. However, they only revealed the social influence on how good would a user evaluate an already-selected object. The social influence on the selection behaviour which is also a crucial reflection of user preference [27, 28], still needs to be investigated. Additionally, while they focused on the nearest predecessor’s influence over the subsequent user, how would the aggregated historical information influence the users’ decision is still an open question.

The present paper aims to distinguish the local-based influence from the global-based influence over the user behaviour of selecting from numerous objects. To achieve this study, one needs to possess the social structure of a collective of people and the records of their sequential selection behaviour over a number of certain objects. Thanks to the development of online systems, data from some websites offers us great opportunities to study the users’ online behaviour. Here we use a large scale data from Yelp.com (see Methods section), where users can not only read reviews on various kinds of businesses such as restaurants, shopping centres, pubs *etc*., but also establish friendships with other users. When a user looking for a business on Yelp, the system offers various kinds of ranking list of businesses on the user homepage, and number of reviews, average rating *etc*. on the business homepage. Those are the global information that could possibly influence a user’s selection decision. On the other hand, there is also a timeline displaying the reviews of his/her friends on businesses which can be recognised as the local information for the user to refer to. Such explicit data provide us the opportunity to explore the question that, whether the opinions of the local neighbourhood, i.e. friends, or that of the whole population matter most for a user to make selection decision?

## Results

**Fig 1:**
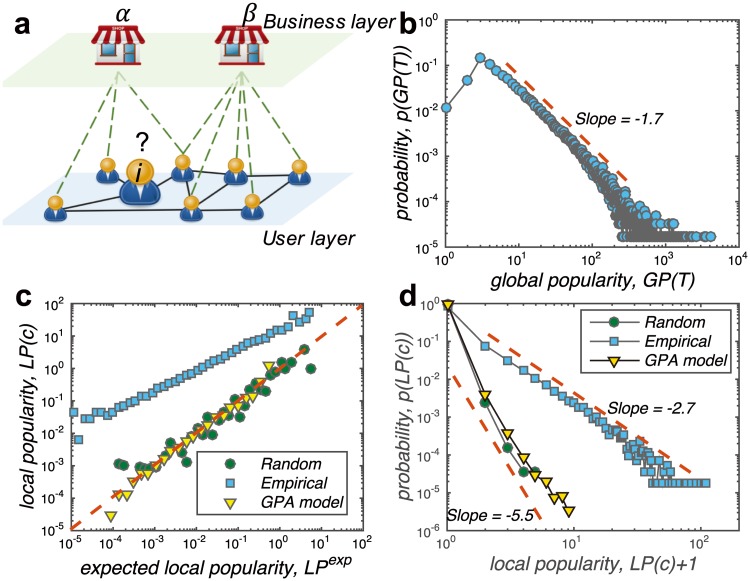
(Color online) **a,** An example of user-business bipartite network with social structure to illustrate the Yelp data set and the research scenario. On the user layer, each user may establish friendships with others and those friends are the target user’s local neighbourhood. On the other hand, the whole user layer is the global environment for each user. The interactions between the user and business layer represented by the bipartite links, are the comment behaviours. Although it is impossible to know exactly each user’s real-world consumption for those businesses, we assume the online comment behaviour could largely reflect what those users have selected (consumed). **b,** The distribution of the businesses’ final global popularity, i.e. popularity at the end *t* = *T* of the Yelp data set, *GP*(*T*). As what have been observed from most networks, the global popularity distribution displays a power-law form with slope of −1.7. **c,** Local popularity of selection behaviours *LP*(*c*) versus the expected local popularity *LP*^*exp*^. The red dashed line shows the condition that *LP*(*c*) = *LP*^*exp*^. While the local popularity of the random experiments and global-driven preferential attachment (GPA) model are very similar to the expected value, the empirical local popularity is significantly higher which suggests that the users tend to select locally popular businesses. **d,** The distribution of real-time local popularity *LP*(*c*). For the empirical data, the local popularity follows the power-law distribution with slope of −2.7. On the other hand, the local popularity of the GPA model being very similar to the random experiment, cannot reproduce the empirical observation.

The Yelp data set applied in this study consists of 1,569,264 comments on 61,184 businesses by 366,715 users. Especially, the explicit social structure of the users’ online friendships is known. For detailed information and descriptive statistics of the data set, see Methods section and Fig A in S1 File. According to the Yelp data set, the scenario of this study could be consequently described by the user-business bipartite network with social structure shown as Fig 1(a). In the user layer, two users will connect with each other if they are friends in Yelp; and between the layers, a user node will connect to a business node if s/he has commented it. Therefore, the local information for a target user *i* can be represented by the opinions and decisions of his/her local neighbourhood, i.e. those users who are connecting to him/her. On the other hand, the global information then is the opinions and decisions of the whole user layer, i.e. all users either connected or unconnected to user *i*.

As the popularity information is the most fundamental signal of social influence which has been argued to be self-reinforcing [29, 30], we take popularity to describe the local and global information in the system. Thusly, the *local popularity* of a business *α* subjecting to a specific user *i* at time *t*, *LP*_*iα*_(*t*), is defined as the number of user *i*’s friends who have connected to the business *α*. The *global popularity* of a business *α* at time *t*, *GP*_*α*_(*t*) is the number of users in the whole user layer who have connected to it. Take the network shown in Fig 1(a) as an example, the local popularity of business *α* and *β* for the target user *i*, *LP*_*iα*_ and *LP*_*iβ*_ are 3 and 1 respectively. The global popularity of business *α* and *β*, *GP*_*α*_ and *GP*_*β*_ are 3 and 5 respectively. Given the fact that the business *β* is globally more popular than *α*, classical preferential attachment mechanism [12, 13, 15] may predict that it is more likely for the target user *i* to connect to the business *β*. However, despite the low global popularity, the business *α* has been connected by all of the user *i*’s three friends. Being locally more popular, the business *α* is more likely to be recommended by the target user’s friends which may also enhance the possibility for user *i* to connect to it. Thusly, a fundamental question the present paper aims to answer could be simplified as: when making selection decisions, it is more likely for a user *i* to be influenced by his/her friends (connect to business *α*), or by the crowd (connect to business *β*)?

### Identifying local-based social influence

Similar to what has been observed from many systems, the global popularity of businesses in Yelp also follows the power-law distribution as shown in Fig 1(b), i.e. *p*(*GP*(*T*)) ∼ *GP*(*T*)^*γ*^ with *γ* = −1.7, where *t* = *T* is the end of the data. Such power-law distribution of the global popularity is normally modelled by preferential attachment mechanism where the popular objects could attract more connections. However, it is still an open question that is the local popularity also attractiveness?

To explore whether is the local popularity attractiveness, here we analyse the real-time local popularity when each behaviour took place. For a connection between user *i* and a business *α* which is established at time *t* = *c*, we examine the real-time local popularity of the business *α* corresponding to the user *i*, *LP*_*iα*_(*c*). In other words, the real-time local popularity is the number of user *i*’s friends who have already connected to the business *α* before s/he did. Suppose a user *i* with *k*_*i*_ friends, connected to a business *α* with global popularity *GP*_*α*_(*c*), the expected local popularity should be $LP_{i\alpha }^{exp}\left(c\right)=k_{i}\cdot GP_{\alpha }\left(c\right)/M$, where *M* is the total number of users in the system. The expected local popularity $LP_{i\alpha }^{exp}$ describes the case that, the target user *i*’s local neighbourhood has no particular preferences in the business *α* when the user *i* connected to it, *i.e*. there is no local-based social influence. However, as shown in Fig 1(c), the empirical local popularity of those selection behaviours is much higher than the expected local popularity. Such result suggests that, businesses which are relatively more popular in the target user’s neighbourhood are more likely to be selected. This is the evidence of the presence of the local-based social influence, that the user may select what his/her friends have selected. Such phenomenon cannot be explained by traditional preferential attachment mechanism because the local-based information has not been considered. The traditional preferential attachment, i.e. the global-driven preferential attachment (GPA), believes that the probability of new connections is determined by the global popularity rather than the local popularity. Consequently, the real-time local popularities *LP*(*c*) generated by the GPA model (S1 File) coincide with that of the random rewiring (S1 File), which is very similar to the expected local popularity. Additionally, the empirical local popularity *LP*(*c*) exhibits a power-law distribution, i.e. *p*(*LP*(*c*)) ∼ *LP*(*c*)^*γ*^ with *γ* = −2.7, as shown in Fig 1(d). On the other hand, the local-popularity distribution of the GPA model and the random rewiring being similar to each other, are in very narrow ranges. Though also exhibit a linear pattern in the log-log plot, the slopes of LP distributions of the random rewiring and GPA model are −5.5.

The analysis indicates that, the local-based social influence is also a driver of users’ selection behaviour in addition to the global-based social influence. Especially, we need to pay close attention on the mechanism of users’ selection decision because the observations cannot be explained by the random mechanism or the traditional preferential attachment mechanism.

### Distinguishing the local-based influence from global-based influence

The power-law distribution of the global popularity *GP*(*T*) implies the fact that, the evolution of the Yelp system could be characterised as ‘rich get richer’ dynamics. Consequently, the global popularity must be driving the users’ selection behaviours. On the other hand, we have also found the local popularity to be a notable driver of the system’s evolution. In other words, a business being either globally popular (large *GP*(*t*)) or locally popular (large *LP*(*t*)) enhances its probability to be selected by users. However, the global popularity and local popularity may be confounding factors because locally popular businesses are likely to be also globally popular. Therefore, here we try to distinguish the influence of local and global popularity on users’ selection behaviour by analysing the probability of a business to be selected *P*(*s*) (Materials and Methods section, S1 File).

**Fig 2:**
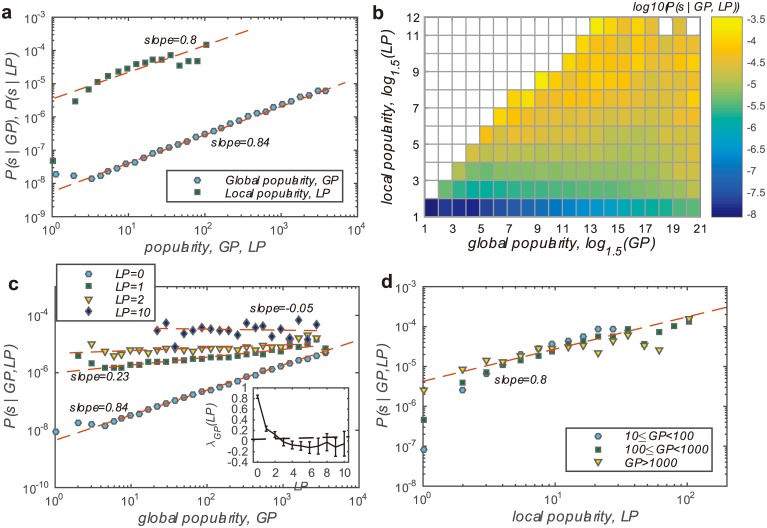
(Color online) **a,** The probability of being selected conditional to the global popularity and local popularity respectively. The results are plotted in log-log scale. The correlations between the probability and the global/local popularity are fitted by functions with form of *P*(*s*|*GP*)∼*GP*^*λ*^ and *P*(*s*|*LP*)∼*LP*^*λ*^. The fitted parameters are *λ*_*GP*_ = 0.84 and *λ*_*LP*_ = 0.8 for the global and local popularity respectively. **b,** A colourmap to describe the probability conditional to global and local popularity, *i.e*. *P*(*s*|*GP*, *LP*). There are blanks on the colourmap and it is because no data records satisfy the the condition that *GP* < *LP*. **c** and **d,** Horizontal and vertical cross sections of the probability shown in subplot *b*, i.e. the conditional probability with LP and GP controlled respectively. One can still fit the slopes of the linear patterns in the log-log scale. The inset of subplot (c) shows the slope of the fitting *λ*_*GP*_(*LP*) versus the control of local popularity *LP*.

We firstly examine the probability of being selected conditional to the global popularity *P*(*s*|*GP*) and the local popularity *P*(*s*|*LP*) separately. As shown in Fig 2(a), both the global and local popularity have positive correlation with the probability. The larger a business’s either global or local popularity is, the more likely it will be selected by users. The positive correlations prove again that both the global- and local-based social influence exist in the selection behaviour of Yelp users. Furthermore, the correlations could be well fitted by the power-law functions, *P*(*s*|*GP*)∼*GP*^*λ*^ and *P*(*s*|*LP*)∼*LP*^*λ*^. The parameter *λ* describes the increase speed of the probability as the business getting more and more popular either globally or locally. One can therefore use the parameter *λ* to quantify the intensity of social influence. As indicated by the results that *λ*_*GP*_ = 0.84 and *λ*_*LP*_ = 0.8, the global-based influence and the local-based influence are of similar intensity.

We further analyse how the global and local popularity jointly influence the probability of being selected *P*(*s*|*GP*, *LP*). One can find from Fig 2(b) that, it is mainly the local popularity determining the probability. A locally unpopular business has very limited chance to be selected by users even if it is globally very popular. The local popularity is more effective in terms of the value of the probability. It can be observed from Fig 2(a) that, *P*(*s*|*GP* = 10^3^) ≈ *P*(*s*|*LP* = 2). To avoid the confounding effect of the global- and local-based social influence, we take the local and global popularity as control by turns. When controlling the local popularity *LP* at a fixed value *LP*_0_, the correlation between the probability *P*(*s*|*GP*, *LP* = *LP*_0_) and the global popularity *GP* could still be well fitted by the power-law functions as shown in Fig 2(c). For businesses with local popularity *LP* = 0, *i.e*. none of the target user’s friends have selected it, the global popularity is able to significantly enhance the selecting probability (*λ*_*GP*_(*LP* = 0) = 0.84). However, even only one of the target user’s friends selected the business (*LP* = 1), the intensity of global-based social influence will drop to a quite low level, *λ*_*GP*_(*LP* = 1) = 0.23. As the local popularity *LP* increases, the global-based social influence vanishes or even changes to weak, negative influence. The inset of the Fig 2(c) indicates that, there is no apparent global-based social influence for cases with *LP* > 2. On the other hand, the local-based social influence is always very significant regardless of the global popularity level as shown in Fig 2(d) and the intensity *λ*_*LP*_(*GP*) ≈ 0.8, ∀*GP*.

Excluding the confounding effect among global- and local-based social influence, we could conclude that, the global-based social influence on selection behaviours exists only if there are not many friends’ opinions to be referred to. It is the local-based social influence always governing the users’ selection behaviour.

### Modelling the global- and local-based social influence

To better understand the mechanisms of the local- and global-based social influence, here we propose an evolutionary model to describe the users’ selection behaviour. The fundamental mechanism of many systems can be described by the preferential attachment [12] where popular nodes have more chances to get new connections. Inspired by the models [15, 16, 30] that have been trying to describe the evolution of bipartite networks based on the preferential attachment, we assume there may exist both the *local-driven preferential attachment* and the *global-driven preferential attachment*.

We consider a system with *N* users with a pre-defined social network among them, and a growing number of objects (in this case, businesses). When each object comes to the system, we suppose it will be connected by a random user. At each time step of the evolution *t*, a user *i* is chosen uniformly at random to connect to an object. With a probability *μ*, the user *i* will connect to the object according to the mechanism of local-driven preferential attachment, and thusly the probability of each object *α* being connected *prob*^*local*^(*α*) is,

$$\begin{array}{c} prob^{local}\left(\alpha \right)=\frac{LP_{i\alpha }\left(t\right)}{\sum_{\beta \in \Gamma_{i}}LP_{i\beta }\left(t\right)}, \end{array}$$ (1)

where Γ_*i*_ is the set of objects that the user *i* has not connected to yet at the time *t*. Accordingly, the user *i* has a probability of 1 − *μ* to perform a global-driven preferential attachment where the probability of each object *α* being connected *prob*^*global*^(*α*) reads,

$$\begin{array}{c} prob^{global}\left(\alpha \right)=\frac{GP_{\alpha }\left(t\right)}{\sum_{\beta \in \Gamma_{i}}GP_{\beta }\left(t\right)}. \end{array}$$ (2)

Combining the local- and global-driven preferential attachment, we have the probability of an object *α* to be connected *prob*(*α*) which reads,

$$\begin{array}{c} prob\left(\alpha \right)=\frac{\mu \cdot LP_{i\alpha }\left(t\right)}{\sum_{\beta \in \Gamma_{i}}LP_{i\beta }\left(t\right)}+\frac{\left(1-\mu \right)\cdot GP_{\alpha }\left(t\right)}{\sum_{\beta \in \Gamma_{i}}GP_{\beta }\left(t\right)}. \end{array}$$ (3)

In the model, *μ* is a tunable parameter ranging in [0, 1] which controls the influence of local- and global-driven attachment. The intensity of the local-based social influence and global-based social influence could therefore be described by the parameter *μ*. The larger the parameter *μ* is, the stronger the local-based social influence would be and at the same time, the weaker the global-based social influence would be.

**Fig 3:**
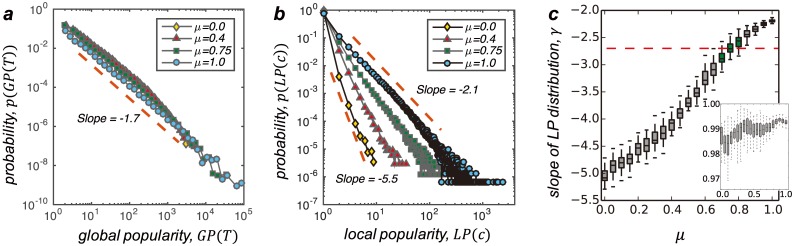
(Colour online) Results of the evolutionary model. With respect to the empirical data, we set the initial state of the simulation same with the data applied in this study, i.e. *M* = 61, 184, *N* = 366, 715 and we use the empirical social structure as the pre-defined network among users. Furthermore, each simulation continues for 1,569,264 steps (same with the empirical data). **a,** Distributions of the simulated global popularity. The simulations with different parameters *μ* can all reproduce the power-law global popularity distribution with slope same to the empirical observation. **b,** Distributions of the real-time local popularity with different parameters *μ*. Each distribution exhibits a linear pattern in the log-log plot. **c,** The slope *γ* of the linear pattern for local popularity distributions with different parameter *μ*. For each parameter *μ*, the result is calculated based on 100 independent simulations. For each simulation, the fitting is based on a linear regression after taking logarithm for the simulated local popularity *LP*(*c*) and the frequency (p.d.f.) of it *p*(*LP*(*c*)) (S1 File). The inset in the subplot (c) shows the coefficient of determination *R*^2^ of corresponding fittings. The *R*^2^ of the fittings are generally larger than 0.98 which indicates that the fittings can be considered good for all the experiments with different parameters *μ*. The red dashed line is the slope *γ* of the empirical local popularity distribution shown in Fig 1(d), i.e. *γ*_*em*_ = −2.7. The green boxes are those which agree with the empirical result.

We use the model to simulate the evolution of the user-business bipartite network with underlying social structure to explore whether the model could reproduce the empirical observations of the local popularity distribution. To avoid the influence of other possible factors, we use the population (of both users and businesses) and the social structure of the Yelp data as the initial configuration of the model (S1 File). As shown in Fig 3, the proposed model can generate power-law distributed global popularity with slope same to the empirical observation regardless of the parameter *μ*. On one hand, such result suggests that, the traditional preferential attachment can indeed explain the emergence of the scaling phenomenon for the popularities. On the other hand, the local-driven preferential attachment may also be a driving mechanism of the power-law distribution. As to the local popularity, though the distributions with different parameters *μ* all exhibit linear pattern in the log-log plot, the slopes are different. For the parameter *μ* = 0 (actually the traditional preferential model, which is totally driven by global popularity), the slope *γ* ≈ −5.5, which is very similar to the random experiments shown in Fig 1(d) where the local-based social influence has been removed. As the parameter of the model *μ* increases, the slope *γ* also gradually increases (Fig 3(b)). For the experiment with *μ* = 1 where the evolution is totally driven by the local-based preferential attachment, the slope could reach *γ* = −2.1. Furthermore, to reproduce the slope of the empirical local popularity distribution, *i.e*. *γ*_*em*_ = −2.7, the parameter of the model should be 0.7 ≤ *μ* ≤ 0.8 as shown by the green boxes in Fig 3(c).

From the point of view of GP distribution, any combination of global-driven preferential attachment and local-driven preferential attachment could explain the empirical findings. However, from the point of view of LP distribution, the local-driven preferential attachment is responsible for about 75% of the evolution. In other words, 75% of the Yelp users’ selection behaviours are driven by the local-based social influence according to the consistency between the evolutionary model and the empirical observations.

## Discussion

The development of the modern world offers us numerous choices when we want to read a book, watch a movie or go out for a dinner. While making choices, the social influence has long been argued to be driving our behaviour. To distinguish the local-based social influence and the global-based social influence is to explore whether it is our friends’ or the whole population’s opinion that matters most for us to make the decision.

By applying a large scale data from Yelp.com, where users could establish friendships with others and look for businesses, we use local popularity *LP*, which is the popularity of a business in the users’ local neighbourhood of friends, and global popularity *GP*, which is a business’s popularity in the whole system, to represent the local- and global-based signal for the social influence. We found the local-based social influence driving the users’ selection behaviour significantly in comparison with the random experiments. Additionally, the local popularity of the selection behaviour *LP*(*c*) follows the power-law distribution, which means the evolution of such system could be described by the local-driven preferential attachment mechanism. On the other hand, while the global-based social influence is significant when the local popularity is low, it vanishes as the local popularity increases. Thusly, the global-based social influence only plays a supplementary role in the dynamics, and drives the evolution only if there are not much local opinions.

The bipartite network is a very efficient model to describe the interactions between users and objects (businesses in this study) [31]. However, the empirical observations in this paper cannot be explained by existing evolution mechanisms. While most of the existing mechanisms are based on the preferential attachment, the local- and global-driven attachments have not been properly distinguished. We proposed an evolution model for bipartite networks combining both the local- and global-driven preferential attachments. With a tunable parameter controlling the intensity of local-based social influence and global-based social influence, the empirical observations were reproduced by the proposed model. The results suggest that, 75% of the connections in Yelp user-business bipartite network are established according to the local-driven preferential attachment mechanism rather than the traditional global-driven preferential attachment mechanism. Despite that the ‘rich get richer’ phenomenon in complex systems has been widely discussed, this may be the first evidence found, to our knowledge, that such phenomenon is actually ‘locally rich get richer’.

While the significant local-based social influence has been found, it is necessary to consider the fact that, friends tend to have similar interests given the homophily is a fundamental phenomenon in the social network evolution [23, 32, 33]. Although all of the connections to the same business among friends are characterised as the local-based social influence, it is actually difficult to know whether it is because one’s selection behaviour influenced another or they just have same taste and made the same decision separately. The global-based social influence may also suffer from similar problem that, whether an object getting more and more popular is because of its popularity attracted more attentions, or just because it is of common interest due to its high quality? Nevertheless, the comparison between global-based social influence and local-based social influence still stands and may help to better understand the evolution of such systems.

The evolution model proposed in the present paper is expandable. While the underlying social structure of the model is pre-defined in this study, it actually could be also evolving and growing. Considering the homophily effect that users with similar interests are more likely to befriend with each other, the user-object interactions may also influence the formation of user-user interactions. Therefore, the mechanism governing the co-evolution of user-object interactions and user-user interactions is still an open question.

## Methods

### Yelp data

The Yelp.com is a website providing reviews and information on various businesses including restaurants, cafes, theatres or even clinics and hospitals. Once registered to the system, users are exposed to both local information and global information of different businesses which makes Yelp an ideal scenario for studying social influence. The Yelp data applied in this study is an open dataset provided by the Yelp themselves, and is accessible in Yelp data challenge website: https://www.yelp.co.uk/dataset_challenge. While they may constantly update the published data, we accessed the data in January 2016. We declare that all the information including the business IDs and user IDs in the downloaded data are encrypted, and we have complied with all the terms of use of the website.

### Probability of being selected

The probability is estimated from the Yelp data, by dividing the number of real selection behaviours by the number of possible selection behaviours.

The number of real selection behaviours, *N*_*RS*_ is calculated by directly adding up the selection records from the data. On the other hand, the number of possible selection behaviours, *N*_*PS*_ is defined by supposing each business may possibly be selected by every user who has not yet selected it in a certain period *δt*. For example, a business *α* has not been selected by three users, *i*, *j* and *k*. The business *α* would have three possible connections for every *δt* until one of the users actually selects it and then have two possible connections for every *δt* sequentially.

The probability *P*(*s*), is then defined as the fraction of the real number and the possible number of selection behaviours conditional to global and local popularity, i.e. *P*(*s*) = *N*_*RS*_/*N*_*PS*_. In the analysis of the present paper, the time interval is *δt* = 1*day*, i.e. we suppose in each day, each business could be possibly selected by every user who has not selected it yet. Note that, to ensure the accuracy of the estimation, we consider the entrance time of the businesses and users. The business and user will be considered in the calculation of the possible selection behaviours only if it/he is already in the system.

A more detailed description and example of the calculation can be found in S1 File.

## Supporting information

S1 File Appendix text to support and further explain the arguments in main text, and Figs A, B, C. (PDF)
