# Supplementary material for: Social influence on selection behaviour: Distinguishing local- and global-driven preferential attachment
Source: PLoS One. 2017 Apr 13;12(4):e0175761. doi: 10.1371/journal.pone.0175761 (PMC5391099; doi:10.1371/journal.pone.0175761)
Supplement: S1 File — (PDF) [file pone.0175761.s001.pdf]

Supporting Information for
Social influence on selection behaviour: distinguishing local- and global-driven preferential attachments

Xue Pan[1], Lei Hou[1] and Kecheng Liu[1,2]

[1] *Informatics Research Center, University of Reading, Reading RG6 6UD, United Kingdom*
[2] *Data Science and Cloud Service Research Centre, Shanghai University of Finance and Economics, Shanghai 200433, China*

Yelp data.

The yelp.com is a business review website where users can check on countless businesses such as restaurants, cafes, theatres, or even clinics and hospitals. Beside of the basic business information such as the address, opening hours, parking etc., users can especially check on others' ratings and reviews on a particular business. After gathering the opinions of others, a user may make his/her own decision accordingly that whether or not to go to the business, or which one to go to. As a consequence, the opinions of others are very likely to influence the decision process of the user. This makes the Yelp an ideal scenario for the studies of social influence on user consumption, selection behaviour, and user preference. Especially, one of the most appealing features of Yelp is its social networking. A user can establish friendships with other users either to be his/her real-word friends or those who write reviews s/he finds trustworthy in the system. On the homepage of the Yelp, there displays the list of your friends' recent activities (reviews) besides the list of non-friends's activities. Therefore, the friends' opinions are also influential factors for a user to make decision. Considering all the features and settings of the Yelp website, we believe it is very suitable for this study to explore the local- and global-based social influence over the interactions between users and objects (businesses).

Yelp, being enthusiastic on scientific research, has published their data and been holding challenges for many years. The data set used in this study was downloaded from Yelp challenge website https://www.yelp.co.uk/dataset_challenge. While they constantly update the published data set, the data in this study was accessed in January 2016. Although quite detailed data is published, we only use the wiring patterns and the timestamps of the system, i.e. which user befriended with which users, and commented which businesses at what time. Therefore, the information we considered from the published data can be perfectly described by the user-business bipartite network with underlying social structure as shown in Figure 1 but with a temporal manner. The date of each review been conducted and each user registered is known, but the time information for the establishment of friendship is unknown in the data. Therefore, for the user-business connections, the timestamps are the exact time provided by the data, while for the user-user connections, the timestamps are estimated as the later date of the two connected users' registrations. In other words, if two users are connected in the data, we consider the connection was established when both of the users had registered to the system.

Random experiment.

To explore whether the observations about the real-time local popularity LP(c) is caused by random mechanism, we compare the results with random experiment. In the random experiment, the global popularity of each business and the whole social structure are unchanged, while the wiring patterns between users and businesses are rewired. For example, if a business α was connected by GP_α users in the original data, we select GP_α users anew from the whole population uniformly at random and let them to connect to the business α. Meanwhile, we also keep the timestamp of each connection. In this way, the local-based social influence would be removed because a user's selections would not be similar to his/her friends' anymore.

Global preferential attachment (GPA) model.

To explore whether the empirical observations could be explained by traditional models, we take traditional preferential attachment mechanism to simulate the evolution of user-business system. Traditional preferential attachment mechanism believes that the global popularity (degree) is the driver of the network evolution, and therefore, we denote such method with Global Preferential Attachment (GPA).

In order to make the simulated results comparable to empirical observations, we take the real size of the network, i.e. we consider a network consists of $N = 366,715$ users and a growing number of businesses. However, as the GPA model considers only the business's global popularity, the underlying social structure is irrelevant to the evolution and thusly not considered in this model. The growing rate of the businesses is set to be the real rate as shown in Figure A (b). When a business enter the system, we suppose it to be connected by a random user, which means the global popularity of each business at its entrance time is $GP = 1$. During the evolution, at each step of the simulation, a user i is randomly selected from the whole population to establish a connection to an existing business α. The business α is selected according to a probability proportional to its global popularity, i.e. the probability of each business α being connected at

time step t is $prob(\alpha, t) = \frac{GP_\alpha(t-1)}{\sum_{\beta\in\Gamma_i} GP_\beta(t-1)}$, where Γ_i is the set of businesses that has not been connected by user i at the time t. The simulation continues until the number of user-business links reach 1,569,264 which is the number of links in the Yelp data.

One may find that, the GPA model is actually the local- and global-driven preferential attachment model with parameter $\mu = 0$.

Probability of bing selected.

The conditional probability of a business being selected at a certain condition Θ, $P(s|\Theta)$ is calculated based on the empirical observation. The condition Θ could be any attributes of the business, but in this study, we only consider the popularity information, i.e. global popularity GP and local popularity LP. The probability is simply calculated as the fraction between the number of connections of the business and the possible connections, $p(s|\Theta) = N_{RS}(\Theta)/N_{PS}(\Theta)$. The real number of selection behaviour $N_{RS}(\Theta)$ is the number of established connections that satisfy the condition Θ. The calculation of the possible number of connections $N_{PS}(\Theta)$ is based on the assumption that each user-business pair could be potentially connected to each other in each time interval δt until the connection established. Figure B gives an example of the calculation using a toy evolution data with 3 users and 2 businesses over 3 days. After calculating the numbers of real and possible connections for all the possible conditions, one can get the probabilities for businesses with a certain condition Θ to be selected in a time interval δt.

The calculation in the present paper is based on the time interval $\delta t = 1day$. We regard the data before 2012 as the base data and start the statistics of $N_{RS}(\Theta)$ and $N_{PS}(\Theta)$ from 1st Jan. 2012 to the end of the data. As shown in Figure A (c), 66% of the data totalling $\sum_\Theta N_{RS}(\Theta) = 1,036,440$ records are considered, which is very abundant for the estimation of the probability. Additionally, the registration time of users is also considered, i.e. a user-business connection is considered possible only if the user and business are already in the system.

Slope fittings in log-log plot.

All the slope fittings in the log-log plot in the present paper are based on the linear regression after taking logarithm for the corresponding two sets of original data. For the simulated local popularity LP distributions, we use linear regression model to fit the correlation between $log(LP)$ and $log(p(LP))$, i.e. $log(p(LP)) = \gamma \cdot log(LP) + c$ where $p(LP)$ is the proportion of user-business connections with a local popularity LP. Considering the local popularity exhibits a heavy tailed power-law distribution which is commonly observed in many complex systems, we ignore the data points with $N(LP) \leq 5$. Then, the fitted coefficient γ is considered to be the slope of the distribution in the log-log plot.

According to the coefficients of determination and the standard errors of the fitting shown in Figure C, the fittings are generally good and therefore the slopes of the power-law distribution can be regarded valid.

Local- and global-driven preferential attachment model.

The aim of the proposed model is to explore that to what extent is the evolution of the system govern by local- and global-based social influence. To make the model more fitted to the empirical data in terms of the initial settings, we take the growing pattens of both the businesses and connections shown in Figure A (b,c) as the model configuration. The simulation is carried out in days according to the empirical data. For each day, there would be $NB(t)$ new businesses and $NC(t)$ new connections coming into the system. Each of the new businesses will be initially connected by a random user. The other new connections will be established between random selected user and businesses selected according to a probability shown in Eq. (3).

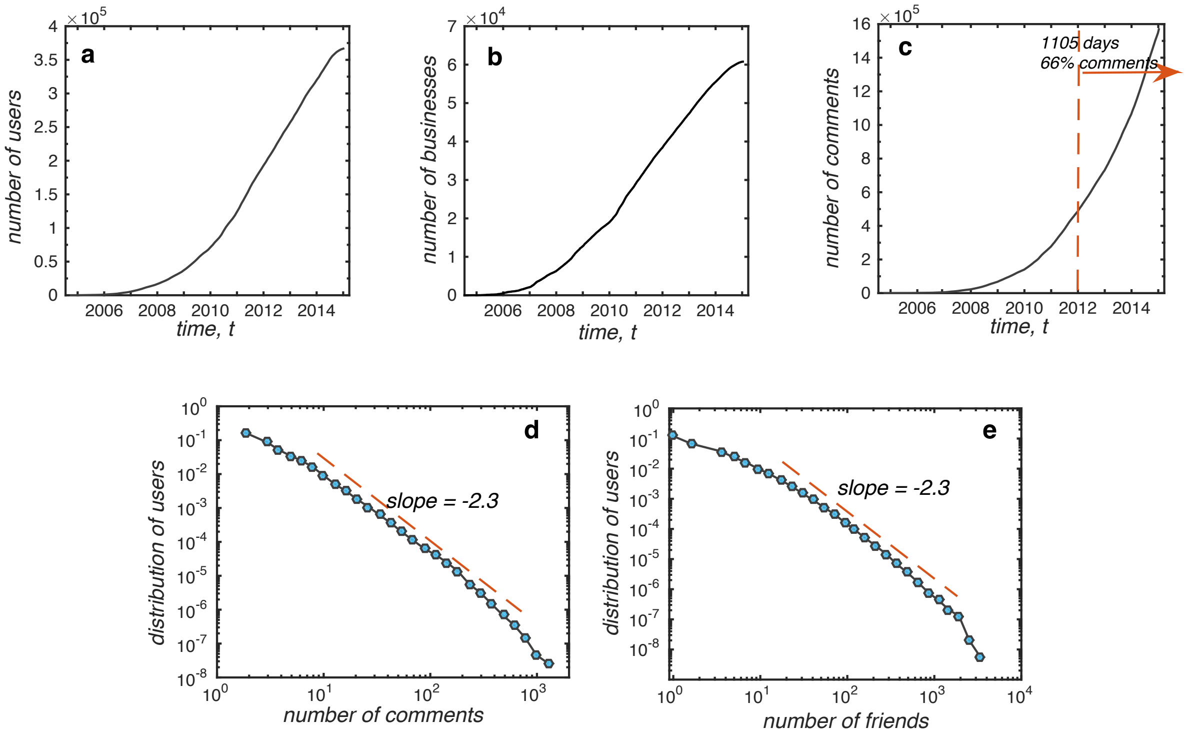

Figure A: **Statistics of Yelp data. a, b, c,** The growth of user population, number of businesses and number of comments respectively in the data over 11 years. Most of the data distributes in the recent years. Note that, while the date of the user registration and user-business connection establishment are given by the data, the time that each business registered to the system is estimated as the first comment date. **d, e,** Distributions of users in terms of number of comments (user-business degree) and number of friends (user-user degree) respectively. The red dashed line in each of the subplots c and d has a slope of -2.3 in the log-log plot.

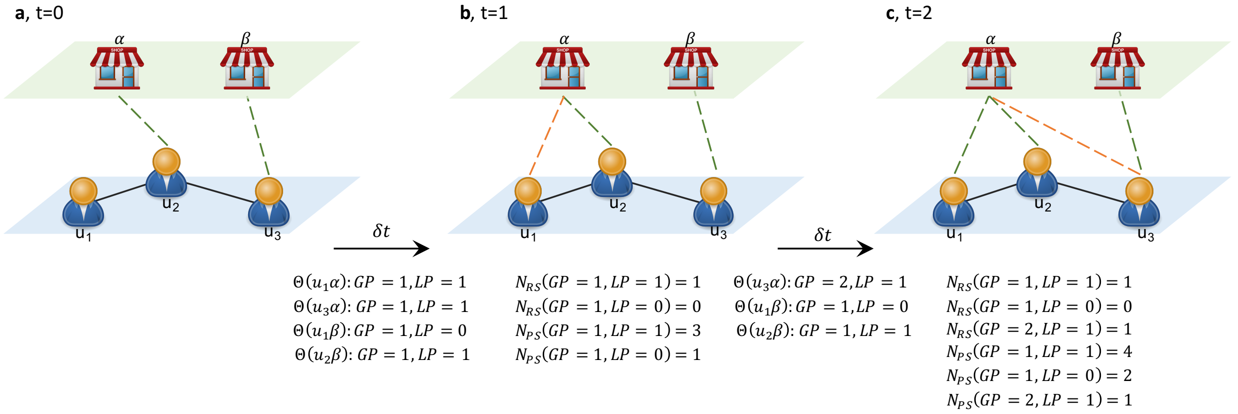

Figure B: **A toy data to illustrate the calculation of probability of being selected,** $P(s)$**.** Suppose a toy data with 3 users and 2 businesses over 3 days. Consequently, there are two time intervals δt, where we should observe the evolution, i.e. from $t = 0$ to $t = 1$ and from $t = 1$ to $t = 2$. During the first time interval, from $t = 0$ to $t = 1$, there are only one connection $u_1 \rightarrow \alpha$ established, while there are in total four possible connections, which are $u_1 \rightarrow \alpha$, $u_3 \rightarrow \alpha$, $u_1 \rightarrow \beta$ and $u_2 \rightarrow \beta$. As the established connection $u_1 \rightarrow \alpha$ is with the condition $\Theta : GP = 1, LP = 1$, we then have $N_{RS}(GP = 1, LP = 1) = 1$. Additionally, among the four possible connections, three are with condition $\Theta : GP = 1, LP = 1$ and one is with condition $\Theta : GP = 1, LP = 0$. As a consequence, the possible numbers are $N_{PS}(GP = 1, LP = 1) = 3$ and $N_{PS}(GP = 1, LP = 0) = 1$. Given that the user u_1 has connected with business α at time $t = 1$, this connection will not take into account for the following possible connections. Similarly we could count the numbers for the second interval from $t = 1$ to $t = 2$. After the statistics, there are in total of 3 conditions appeared in this toy data, i.e. $\Theta_1 : GP = 1, LP = 0$, $\Theta_2 : GP = 1, LP = 1$ and $\Theta_3 : GP = 2, LP = 1$. Therefore, the probability of business with a certain condition Θ, $P(s|\Theta)$ is estimated accordingly as $P(s|GP = 1, LP = 0) = 0/2 = 0$, $P(s|GP = 1, LP = 1) = 1/4 = 0.25$ and $P(s|GP = 2, LP = 1) = 1/1 = 1$. Although there are only limited possible conditions Θ in this toy data, the estimations in the main text would be much more accurate due to the abundant data amount. But the estimations of some extreme conditions such as very large LP and GP will be still inaccurate because such conditions may occur only for limited times.

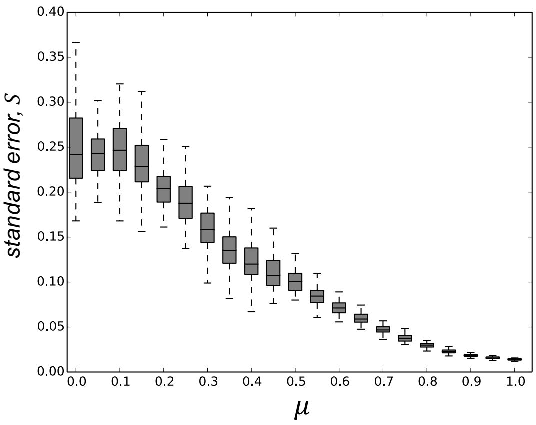

Figure C: **The standard errors S of the fittings for the simulated local popularity distribution.** The results of standard error suggests that the fightings are good except for those with very small parameters μ.
